# Population Genomic Analysis of *Mycoplasma bovis* Elucidates Geographical Variations and Genes associated with Host-Types

**DOI:** 10.3390/microorganisms8101561

**Published:** 2020-10-10

**Authors:** Roshan Kumar, Karen Register, Jane Christopher-Hennings, Paolo Moroni, Gloria Gioia, Nuria Garcia-Fernandez, Julia Nelson, Murray D. Jelinski, Inna Lysnyansky, Darrell Bayles, David Alt, Joy Scaria

**Affiliations:** 1 Department of Veterinary and Biomedical Sciences, South Dakota State University, Brookings, SD 57007, USA; roshanzhc@gmail.com (R.K.); Jane.Hennings@SDSTATE.EDU (J.C.-H.); nuria.garcia@sdstate.edu (N.G.-F.); Julie.nelson@sdstate.edu (J.N.); 2 South Dakota Center for Biologics Research and Commercialization, Brookings, SD 57007, USA; 3 P.G. Department of Zoology, Magadh University, Bodh Gaya, Bihar 824234, India; 4 USDA/ARS/National Animal Disease Center, Ruminant Diseases & Immunology Research Unit, Ames, IA 50010, USA; karen.register@usda.gov; 5 Quality Milk Production Services, Animal Health Diagnostic Center, Cornell University, 240 Farrier Road, Ithaca, NY 14850, USA; pm389@cornell.edu (P.M.); gg363@cornell.edu (G.G.); 6 Dipartimento di Medicina Veterinaria, Via dell’Università, Università degli Studi di Milano, 6, 26900 Lodi LO, Italy; 7 Department of Large Animal Clinical Sciences, University of Saskatchewan, Saskatoon, SK S7N 5A2, Canada; murray.jelinski@usask.ca; 8 Division of Avian Diseases, Kimron Veterinary Institute, Beit Dagan 50250, Israel; innal@moag.gov.il; 9 USDA/ARS/National Animal Disease Center, Infectious Bacterial Diseases Research Unit, Ames, IA 50010, USA; darrell.bayles@usda.gov (D.B.); david.alt@usda.gov (D.A.)

**Keywords:** *Mycoplasma bovis*, bison, pangenome, whole genome sequencing, vaccine, IS element

## Abstract

Among more than twenty species belonging to the class Mollecutes, *Mycoplasma bovis* is the most common cause of bovine mycoplasmosis in North America and Europe. Bovine mycoplasmosis causes significant economic loss in the cattle industry. The number of *M. bovis* positive herds recently has increased in North America and Europe. Since antibiotic treatment is ineffective and no efficient vaccine is available, *M. bovis* induced mycoplasmosis is primarily controlled by herd management measures such as the restriction of moving infected animals out of the herds and culling of infected or shedders of *M. bovis.* To better understand the population structure and genomic factors that may contribute to its transmission, we sequenced 147 *M. bovis* strains isolated from four different countries viz. USA (*n* = 121), Canada (*n* = 22), Israel (*n* = 3) and Lithuania (*n* = 1). All except two of the isolates (KRB1 and KRB8) were isolated from two host types i.e., bovine (*n* = 75) and bison (*n* = 70). We performed a large-scale comparative analysis of *M. bovis* genomes by integrating 103 publicly available genomes and our dataset (250 total genomes). Whole genome single nucleotide polymorphism (SNP) based phylogeny using *M.* *agalactiae* as an outgroup revealed that *M. bovis* population structure is composed of five different clades. USA isolates showed a high degree of genomic divergence in comparison to the Australian isolates. Based on host of origin, all the isolates in clade IV was of bovine origin, whereas majority of the isolates in clades III and V was of bison origin. Our comparative genome analysis also revealed that *M. bovis* has an open pangenome with a large breadth of unexplored diversity of genes. The function based analysis of autogenous vaccine candidates (*n* = 10) included in this study revealed that their functional diversity does not span the genomic diversity observed in all five clades identified in this study. Our study also found that *M. bovis* genome harbors a large number of IS elements and their number increases significantly (*p* = 7.8 × 10^−6^) as the genome size increases. Collectively, the genome data and the whole genome-based population analysis in this study may help to develop better understanding of *M. bovis* induced mycoplasmosis in cattle.

## 1. Introduction

*Mycoplasma bovis* is a member of the class Mollicutes, representing the simplest, wall-less, self-replicating bacterium, known to cause multiple diseases including pneumonia, arthritis and confer late-term bovine abortions [1]. During the course of evolution, the genus *Mycoplasma* underwent regressive evolution and maintained the minimal set of biosynthetic genes necessary for its survival and adjusted to the parasitic lifestyle [2]. *M. bovis* can infect a wide variety of hosts such as cattle, bison, deer and goats and shows tissue specificity within these hosts [3,4]. The first *M. bovis* strain was isolated in the US in 1961 from milk obtained from a dairy herd severely affected with mastitis [5], and it is speculated that the subsequent transportation of infected animals led to its spread across Europe, China, Australia and Israel [6]. This pathogen has been reported to successfully evade the host immune system through the attachment mediated by variable surface proteins (Vsps) along with the biofilm formation [7,8]. For these reasons, *M. bovis* is a significant threat to animal health worldwide and is responsible for substantial economic loss [9,10]. In order to control *M. bovis*, the New Zealand government recently approved culling of large number of cows with the hopes of eliminating *M. bovis* from the island nation, causing an economic loss of hundreds of millions of dollars [11]. Worldwide, *M. bovis* still remains a threat to the dairy industry even after 59 years since its first isolation.

Altogether, it has been well established that the *M. bovis* infection is a multifactorial process and adhesion is the key step towards infection and colonization, mainly mediated through Vsps, which has the ability to undergo substantial antigenic variation involving high-frequency phenotypic switching [12,13]. Therefore, to understand the extended genetic repertoire of Vsps and to gain deep insight into the phylogeny and genomic attributes, we performed a comparative genomic analysis of 250 *M. bovis* isolates, representing the largest genomic dataset studied so far. To this end, we integrated 103 *M. bovis* genomes and one *M. agalactiae* from the public repositories to our dataset of 147 *M. bovis* isolated between 1994 and 2019 from four different countries and four different hosts. This study provides detailed insight into the phylogenetic relationships, virulence profiles, insertion sequences and functional attributes of core and pan genomes of the *M. bovis* isolates.

## 2. Materials and methods

### 2.1. Genomes Included in the Comparative Analysis

Altogether, we sequenced 147 *M. bovis* isolates from four different countries and four different hosts. We downloaded 103 publicly available *M. bovis* genomes from NCBI for comparative analysis with our dataset. Therefore, the total dataset used for comparative analysis is 250 *M. bovis* genomes and one outgroup *Mycoplasma agalactiae* (Table S1). The *M. bovis* isolates sequenced in this study were grown in pleuropneumonia-like organism (PPLO) medium supplemented with 10% horse serum (Thermo Fisher Scientific, Waltham, MA, USA) at 37 °C for 48–72 h. The genomic DNA was isolated using DNeasy Blood & Tissue kit (Qiagen, Germany) according to the manufacturer instructions and quantified using Qubit Fluorometer 3.0 (Invitrogen, Carlsbad, CA, USA). The whole genome sequencing was performed by the Illumina MiSeq sequencer using paired-end V3 chemistry. The datasets for six isolates named NADC83, NADC81, NADC72, NADC53, NADC51 and KRB1 were obtained from the National Animal Disease Center, Ames, IA. These isolates were sequenced using both PacBio and Illumina platforms. PacBio sequencing was carried out by the Yale Center for Genome Analysis (New Haven, CT, USA). Following fragmentation and end repair of genomic DNA, BluePippen size selection was used to enrich for 10–20 bp fragments. Libraries were sequenced using a single SMRTcell per isolate on a PacBio RS II instrument using P6-C4 chemistry. Illumina sequencing (MiSeq; Illumina, San Diego, CA, USA) was carried out at the National Animal Disease Center using 2 × 150 paired-end libraries, prepared with a Nextera XT DNA library preparation kit (Illumina), as detailed in the Reference Guide. The MiSeq 2 × 300-bp paired-end reads for 16 isolates (NADC5, NADC15, NADC24, NADC28, NADC30, NADC32, NADC44, NADC45, NADC50, KRB5, KRB6, KRB7, KRB8, KRB9, KRB10 and KRB11) were provided by Dr. Murray Jelinski. Thereafter, we included eight complete *M. bovis* genomes (08M, Ningxia-1, CQ-W70, HB0801, Hubei-1, NM2012, JF4278 and PG45) from the NCBI database. We also downloaded genome datasets for 95 *M. bovis* strains from the Sequence Read Archive (SRA), based on the availability of metadata information on or before March 31^st^, 2019 (Table S1). Therefore, a total of 250 *M. bovis* genomes were included in this analysis. The outgroup *M. agalactiae* strain 5632 was selected based on genome distance calculation using pairwise average nucleotide identity (ANI) [14].

### 2.2. Genome Assembly and Validation

The raw reads were assembled into contigs using Unicycler [15] that builds an initial assembly graph from short reads using SPAdes 3.11.1 [16], followed by read correction using pilon [17]. The advantage of using Unicycler is that it can also assemble a combination of short and long reads, with high levels of accuracy. The assembled contigs (>500 bp) were validated using QUAST [18]. All assemblies include 200 or fewer contigs and have an N50 of ≥10,000.

### 2.3. Genome Annotation, Core and Pangenome Analysis

All these assembled genomes were annotated using Prokka [19]. A manually annotated reference *M. bovis* (PG45, Hubei-1, HB0801, CQ-W70, NM2012) genbank file was downloaded (https://www.ncbi.nlm.nih.gov/genome/browse#!/prokaryotes/mycoplasma%20bovis) and formatted to a prokka database file format. Open reading frames (ORFs) were predicted and annotated using prokka (-gcode 4) against the formatted database [19]. The general feature format (.gff) files from prokka were used as input for Roary pipeline (percentage identity ≥90) [20] to generate the core genome alignment using PRANK [21]. This was then used to predict the polymorphic sites using snp-sites package [22]. Then, the output was used for model testing using the package modeltest-ng (https://github.com/ddarriba/modeltest). The tree was constructed using Generalized Time Reversible (GTR) + G4 model in RAxML [23].

Further, the orthologs were redefined using orthoMCL [24] available in GET_HOMOLOGUES software package given the following parameters: percentage identity ≥90% and query coverage of ≥75%, using GenBank files. The orthologs thus obtained were annotated using the RAST server using genetic code 4.

### 2.4. Phylogeny Reconstruction and Functional Analysis

In this study, we inferred phylogeny based on whole genome SNPs using kSNPv3.0 [25] with a k-mer length of 31. The SNP-matrix file was used for model testing using the package modeltest-ng (https://github.com/ddarriba/modeltest). GTR + G4 was the best scoring model predicted and this substitution model was used in RAxML [23] to construct the maximum-likelihood (ML) tree. The phylogenetic tree was visualized using GrapeTree [26].

To functionally characterize the *M. bovis* genomes, the amino acid sequences were searched against the eggNOG database [27]. The length of query protein was set to at least 50% in order to be involved in the alignment. The resulting KEGG annotations (KO identifiers) assigned to amino acid sequences of individual isolates were parsed in R (R Core Team, 2018) to generate the abundance matrix. This matrix was used in MeV to construct the gene tree using Pearson correlation and hierarchical clustering [28]. This was then visualized using interactive Tree of Life (iTOL) [29].

### 2.5. Identification of Host-Associated Genes

We applied Scoary to identify genes significantly associated with particular traits, such as host species [30]. This package uses gene presence/absence matrix from Roary and combines Fisher’s exact test, a phylogeny-aware test and an empirical label-switching permutation analysis. The genes that were most significantly associated with a trait, either positively or negatively, were sorted based on *p* adjusted values using multiple test correction. The parameters used for Scoary were *p*-value cut-off <0.05. The protein sequences of the genes identified at this level were used to query the NCBI database to assign proper gene function [31].

### 2.6. Identification of vsp Genes and Insertion Sequences (IS)

To identify *vsp* genes, we used a 70 bp highly conserved nucleotide sequence found immediately 5′ of the start codon in all *vsp* genes as a reference and formatted the database. All the genomes were then searched against this database using the parameters: query coverage 85% and percentage identity 85%. The heatmap was constructed using Euclidean distance and Ward linkage method.

For IS elements, we extracted the Prokka-annotated IS element sequences (*n* = 2640) present in 250 *M. bovis* isolates. Along with this, we included the ISfinder database sequences (*n* = 5685) updated on 25 July 2018. Cd-hit was then used to cluster the sequences using 50% query coverage and 95% sequence identity [32]. At the end, 4878 sequences were used to format the database. Individual genomes were searched against the formatted database using 70% query coverage and 95% sequence identity to identify IS elements. Finally, the comparative abundance matrix of IS elements was generated for the *M. bovis* isolates and visualized in R using the package ComplexHeatmap (R Core Team, 2018).

## 3. Results

### 3.1. Genome Sequencing and General Genomic Attributes of Geographically Diverse M. bovis Isolates

In this study, we included the sequence of 147 *M. bovis* genomes isolated between 1994 and 2019 from four different countries viz., USA (*n* = 121), Canada (*n* = 22), Israel (*n* = 3) and Lithuania (*n* = 1) Table S1). However, one isolate namely F148 was isolated in Israel from the calves imported from Lithuania and there is a chance that the infection may have arisen during the transport. Most of these isolates were mainly contributed by two host types namely bovine (*n* = 75) and bison (*n* = 70). Out of 147 *M. bovis* genomes, four (NADC 51, NADC72, NADC 81 and NADC83) were assembled into single contigs. We then added eight complete and 95 reassembled draft *M. bovis* genomes from the NCBI database, for which at least partial metadata information was available (Table S1). Altogether, a total of 250 *M. bovis* genomes were analyzed in this study, by far the largest whole genome dataset for *M. bovis* isolates. Based on origin, these isolates were distributed across seven countries: the United States (56%), Australia (30.8%), Canada (8.8%), China (2.4%), Israel (1.2%), Lithuania (0.4%) and Switzerland (0.4%) (Table S1). Bovine genomes constituted the largest dataset representing 71.2% (*n*= 179) of genomes, followed by bison isolates (28%; *n* = 70) and one isolate each from mule deer and white tail deer. Most of these isolates were isolated from either the lung (36.05%, *n* = 53) or milk (22.45%, *n* = 33).

The assembly statistics revealed an average genome size of 0.93 ± 0.034 Mbp, with largest genome size in case of strain NADC53 and smallest in case of strain VDL100 (range: 0.839 to 1.071 Mbp). The average GC content was 29.49%, with a maximum and a minimum of 30.27% for Mb61 and 29.25% for NADC53, respectively. The average number of coding sequences was 770 (range: 882–694). When complete genomes of *M. bovis* were analyzed, we find that there as many as 68 ISs with a total size of approximately 0.085 Mbp/genome, suggesting a high level of genomic rearrangement within the genomes of *M. bovis* isolates (Table S1).

### 3.2. Phylogenetic Structure of M. bovis Isolates

**Figure 1:**
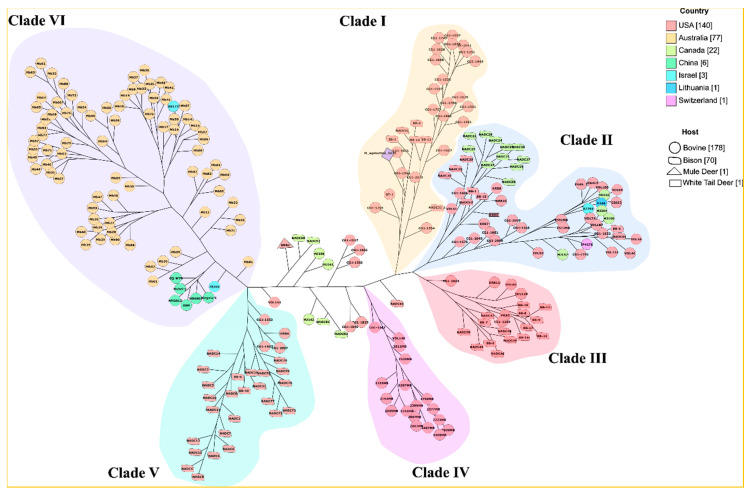
Whole-genome SNP-based phylogenetic tree of 250 *M. bovis* strains rooted to *M. agalactiae* 5632 generated by kSNP3.0. The SNP-matrix file was used for model testing using the package modeltest-ng. Thereafter, the maximum likelihood tree was constructed using Raxml-ng (GTR+G4 model). The tree was visualized using GrapeTree. The color code represents their country of isolation.

Widely used methods to determine the evolutionary relationship among members of the genus *M. bovis* are multilocus sequence typing (MLST) and multiple-locus variable-number tandem repeat (MLVA) [33,34,35]. In this study, we implemented whole genome single nucleotide polymorphism (SNP) method to infer the phylogeny. To resolve this, we used *M. agalactiae* strain 5632 as an outgroup, because this species is the closest (<83.85% at ANI level) relative of *M. bovis*. Using the SNP-based phylogeny, the *M. bovis* isolates formed six different clades (Figure 1). Based on geographical location, the Australian isolates formed a separate clade (clade VI) along with all the Chinese (CQ-W70, Hubei-1, NM2012, 08M, HB0801 and Ningxia-1) and two Israeli (strain 78204 and 88172) isolates (Figure 1). In contrast to this, the USA isolates showed a high degree of genomic divergence and thus clustered in five different clades (clade I-V). Out of five clades, four (I, III, IV and V) were exclusively occupied by the USA isolates (Figure 1). This could be because of sampling bias as nearly 56% of the isolates originated from the USA. However, 15 Canadian isolates and one each from Switzerland (JF4278), Israel (87793) and Lithuania (F148) clustered with the USA isolates in clade II (Figure 1). For *M. bovis* isolates, strain CG1-1544 from clade I clustered with the outgroup *M. agalactiae* strain 5632, showing the highest degree of ancestral relationship. Clade IV harbored all bovine isolates, while clades III and V were dominated by bison isolates. However, there was no clear-cut clustering pattern based on host origin. The isolate from white tail deer (KRB8), clustered with bovine isolates in clade II. In contrast to this, a mule deer isolate (KRB1) did not fall in any major clade, but instead clustered with the bison isolates NADC98 and NADC97. There is no apparent epidemiologic link between KRB1(a 2012 isolate from Nevada), and NADC97 and NADC98, which were obtained in 2017 from different anatomic sites of a single bison in Alberta.

### 3.3. Core and Pangenome Analysis

**Figure 2:**
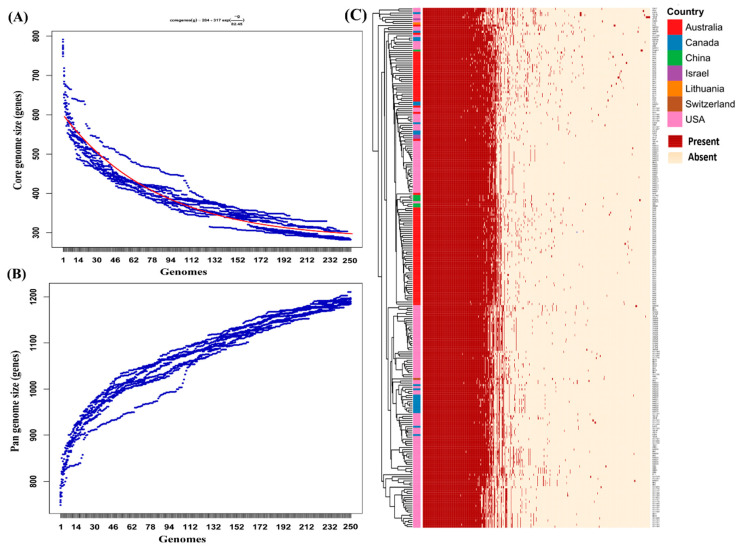
The estimation of core genome and pan genome structure of *M. bovis* isolates (**A**) & (**B**) represents the core genome (*n* = 283) plot with Tettelin fit and Pan genome (*n* = 1186) estimated using get_homologues at a query coverage and percentage identity of 75% and 90%, respectively. (**C**) The gene presence-absence matrix of the pangenome from Roary was clustered using correlation distance and average linkage method.

The core and pangenome analysis revealed the presence of 283 and 1186 coding genes across 250 *M. bovis* isolates, respectively. The high number of accessory genes observed in pangenome suggested that the pangenome is still not fully complete and the addition of more *M. bovis* genomes will increase the pangenome size (Figure 2). The conserved 283 coding sequences span across 40 subsystems with an average size of 0.248 Mb. A majority of these genes code for ribosomal (*n* = 45; 15.9%) and hypothetical (*n* = 51; 18.02%) proteins. However, the core genome was also found to harbor genes associated with virulence. A 454 amino acid long *α*-enolase (phosphopyruvate hydratase) protein was conserved in the core genome of M. bovis. This protein is known as a cytosolic metalloenzyme responsible for the conversion of 2-phosphoglycerate into phosphoenolpyruvate [32]. But, this protein in case of M. bovis has been reported as a surface exposed protein that helps M. bovis to adhere to embryonic bovine lung cell lines with the help of host plasminogen [33]. Therefore, the presence of this protein in the core genome could be of great importance that establishes the invasion of M. bovis within the host tissue [33,34]. A 454-amino acid size (Fi M. bovis. Two lipoate-protein ligase A (*lplA*) genes were also identified in the core genome. Inte. restingly, *lplA* genes were also identified as potential virulence factors [36,37].

Apart from this, the ATP-Binding Cassette (ABC) superfamily proteins were also abundant in the core genome. A complete set of genes for an ABC uptake system i.e., the *oppABCDF* transporter, was conserved across all the *M. bovis* isolates. This system is well known to transport peptides that not only help in cell nutrition [38], but also affects the cell viability [39]. In addition to this, the spermidine/putrescine importer genes *potA*, *potB* and *potC* were also present in the core genome. Altogether, the dominant subsystems in the core genome were related to protein metabolism, nucleosides and nucleotides, carbohydrates, RNA and DNA metabolism, genes responsible for fatty acids, lipids and the isoprenoides subsystem were not present, suggesting that *M.bovis* depends on the host for their production.

### 3.4. Function-Based Clustering and its Implication for Vaccine Development

**Figure 3:**
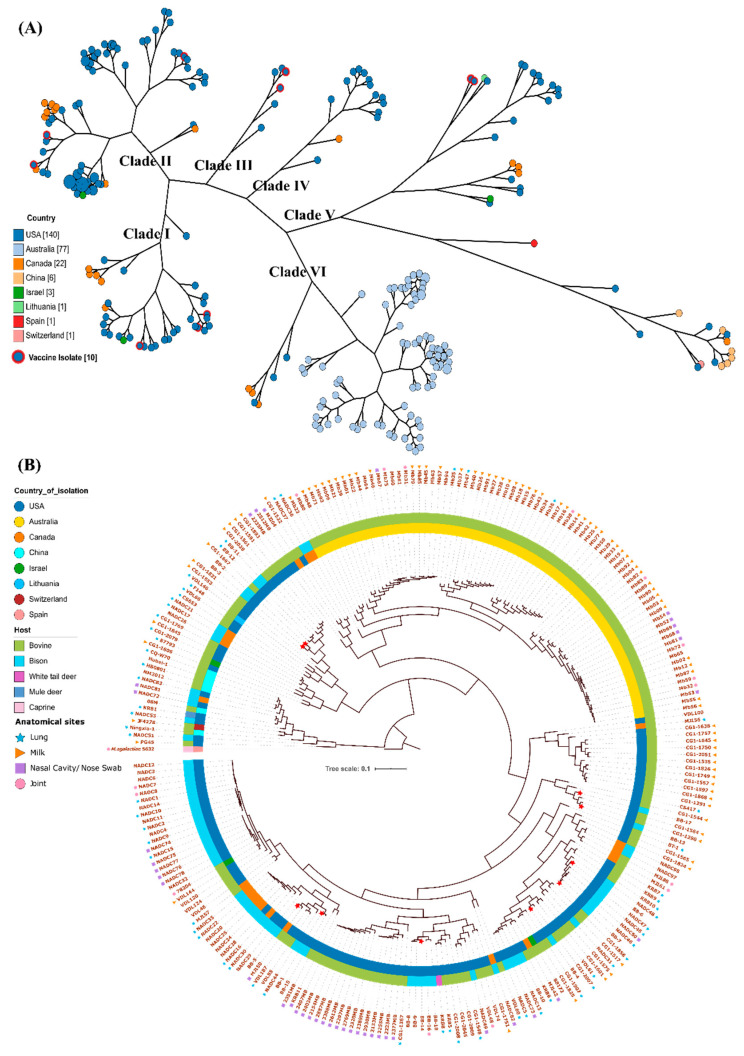
Function-based clustering of *M. bovis* isolates. The kegg matrix was used in MeV to construct the gene tree using Pearson correlation and hierarchical clustering. The tree was visualized using (**A**) GrapeTree (green circles represent the vaccine isolates) and (**B**) iTOL: The outer and inner ring represent the host and country from which they were isolated, respectively. The red star symbols in the branch depicts those strains that are considered for use in autogenous vaccines.

Preventive herd health has become an utmost priority for livestock management and vaccination is a proven backbone for this program. Based on federal regulations, autogenous vaccines must be comprised of strains isolated in conjugation with the animal disease. Therefore, in this study we have included sequence comparisons of ten isolates used as an autogenous vaccine for bison: BB-1, BB-2, BB-3, BB-4, BB-5, BB-6, BB-7, BB-13, BB-14 and BB-17. All were isolated from bison and originated from five different herds in the United States. To assess the degree of functional variation of this blend, we performed function-based clustering (Figure 3). Based on function, we obtained six major clades and interestingly all the Australian isolates clustered together in cluster VI (Figure 3A). All ten vaccine isolates represent four clades: three members each from clades I and II, and two members each from clades III and V (Figure 3A). The clustering pattern of autogenous vaccine isolates suggests that although they are functionally diverse, there are no representatives of certain clades or sub-clades (Figure 3B). In contrast, vaccine isolates within the same lineage sometimes cluster together in close proximity. For example, BB-2/BB-3 in clade V and BB-13/BB-17 in clade III show maximum functional similarity and hence cluster together. Similarly, BB-6/BB-7 (clade I) and BB-5/BB-1 (clade II) are functionally related. Therefore, in order to generate a more broadly effective autogenous vaccine it may be necessary to consider isolates from those clades for which there is no representative strain available in the blend.

### 3.5. Vsp Dynamics and IS Elements

**Figure 4:**
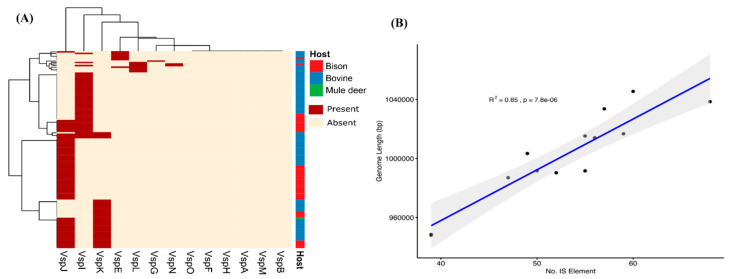
(**A**) The abundance of *vsp* genes across the *M. bovis* isolates used in this study. The *vsp* genes from strain PG45 were used to query the *M. bovis* isolates using a query coverage and percentage identity of 75% and 90%, respectively. Both rows and columns are clustered using Euclidean distance and Ward linkage (**B**) Scatterplot of genome length and number of IS elements. The *p* value corresponds to the Pearson Correlation coefficient and the R^2^ is the regression line fit. Shaded area is the 95% confidence interval.

A family of *vsp* genes is known to generate a high degree of surface antigenic variation through recombination and thus provides remarkable phenotypic and genetic flexibility [40,41]. Initially, when we used the 13 *vsp* gene sequences of strain PG45 as a query and searched against the rest of the isolates, none of the isolates were found to harbor the complete set of these *vsp* genes. The most abundant members of this family were *vspJ* (*n* = 73), *vspI* (*n* = 47) and *vspK* (*n* = 37) (Figure 4A). But, when we used the highly conserved 70 bp sequence present immediately upstream of the *vsp* gene start codons as a query, the number of hits was comparatively high. Out of 250 isolates, 29 possessed ≥10 hits, suggesting a high degree of distinctiveness in *vsp* genes at the sequence level (Table S1). In 13 complete genomes, the number of 5′-upstream sequence hits varied from 0 to 14, with 14 in the case of NADC72, and 13 each in NADC81and PG45. No hits were present in the genomes of CQ-W70 and Hubei-1. Among the autogenous vaccine isolates, the number of 5′-upstream sequence hits varied between 2 and 5, suggesting the inclusion of isolates with a high number of *vsp* genes in the autogenous vaccine. Further, the sequence level comparison of these *vsp* genes will be crucial in determining the strain specificity.

Using a similar approach, we investigated the abundance of IS elements in the genomes of the *M. bovis* isolates included in this study. The genome-wide search revealed the presence of a wide variety of IS elements present in the genome. Using match criteria of 70% query coverage and 95% sequence identity, we observed that many isolates have IS elements with a shorter length, i.e., less than 70% query coverage, but in high abundance. Nevertheless, the average number of IS elements in the complete genomes (N50 > 900,000) and draft genomes (N50 > 900,000) of *M. bovis* isolates is 52.76 and 7.215, respectively. This clearly suggests the sequencing methods and computational tools used are unable to completely resolve the ambiguities of highly repetitive regions [42]. Further, we tested the hypothesis that genome size is correlated with the number of IS elements (N50 ≥ 900,000). Our analysis revealed that the number of IS elements found per isolate increases significantly (*p* = 7.8 × 10^−6^) as the genome size increases (Figure 4B).

### 3.6. Gene Level Prediction of Host-Associated Genes

We applied Scoary to carry out pan-GWAS analysis based on gene presence-absence and the host type, i.e., bovine versus bison, to predict the genes associated with host types (Table 1). Using this approach, we predicted genes that were significantly associated with host types (*p* < 0.05), although the high end of pairwise comparison *p* value range exceeds 0.05 (Table 1). We identified numerous host-associated gene clusters, but most of them are predicted to encode either hypothetical proteins or lipoproteins. Interestingly, a gene coding for variable surface lipoprotein was associated with bovine isolates (*p*-adjusted value = 0.0005). Further experimental validation is required to understand its potential role in host adaptation.

## 4. Discussion

In the present study, we investigated the global population structure of *M. bovis* using the largest genome dataset studied so far. The Australian isolates represent 30.8% of the total population in this study, but their phylogenetic distribution is confined to a specific clade (clade VI) (Figure 1), suggesting a high level of genetic similarity. These results are similar to those of Parker et al. [43], who reported that the overall evolutionary changes observed in Australian isolates at the genetic level are minimal despite variations in anatomical sampling sites, geographical location, disease status and time of sample collection. In contrast to this, the USA isolates representing 56% of the total population are distributed among five different clades, i.e., clades I-V, revealing a high level of genomic variation. Similar to this, the Canadian isolates (8.8% of the total population) have a high level of genetic variation and their clustering with USA isolates [44] (Figure 1) suggests their origin and spread occurred through the movement of cattle from USA herds. The Chinese isolates cluster with Australian isolates, indicating their probable introduction from Australian calves, previously reported using other sequence typing methods [45]. Interestingly, the three Israeli isolates included in this study have two different probable origins; isolates 78204 and 88172 appear to have been originated from Australian calves [46], while isolate 87793 is most closely related to isolates from USA calves indicating a North American origin. The SNP based phylogeny suggests that the *M. bovis* isolates shows a mixed pattern of distribution and one reason for this could be the movement of cattle across countries, similar to the international spread of *M. mycoides* subspecies *mycoides*, a pathogen known to causes contagious bovine pleuropneumonia [47,48].

In spite of the lack of divergence in Australian isolates, the core genome represents only 36.8% of the total genes whereas the number of genes in the pangenome was 1186 as defined here for the genus. The size of the core genome and pangenome fluctuates for any genus depending upon factors such as the percentage identity and query coverage cut-offs values used, the numbers of genomes included, the degree of genomic similarity among the strains and any sampling biases within the taxa [49]. Altogether, the pangenome analysis revealed that the size of the pangenome is increasing steadily with the addition of each genome, suggesting that *M. bovis* has an open pangenome that will expand further by the addition of new genomes.

Our analysis found that the *α*-enolase protein was conserved in the core genome of *M. bovis*. Although this protein is present in a variety of prokaryotic and eukaryotic organisms [50,51], it is considered to be a virulence factor in *M. bovis*, playing a role in host cell attachment with the help of host plasminogen [52]. Similarly, two *lplA* genes which have been previously described as potential virulence factors in *M. bovis* [36,37] are present in the core genome. In *Listeria monocytogenes*, defective LplA protein has been associated with abortive growth along with virulence reduction by 300-fold [53]. Therefore, the presence of genes associated with virulence in the core genome helps the organism in adjusting to their ecological niche [36,37,52,54]. In addition, the *oppABCDF* transporter and spermidine/putrescine importer genes (*potA*, *potB* and *potC*) in the core genome help the organism to transport peptides essential for cell nutrition and viability [38,39], and function in cell proliferation and differentiation, respectively [55,56].

Our study also describes a functional genomics-based analysis of a multivalent autogenous vaccine comprised of isolates from multiple clades. Using the KEGG annotations pipeline [27], the formulations could be derived by selecting isolates with functional heterogeneity. In the case of *M. bovis*, genotypic differences have been observed among isolates from a single herd and even at different anatomical sites within the same animal [57,58]. We assumed that implementing traditional empirical approaches to screen individual isolates for vaccine development is time-consuming when considering the difficulty of cultivating and maintaining a large number of isolates in the laboratory. Therefore, for a pathogen like *M. bovis,* where the mechanism of pathogenesis is under study, implementing a functional genomics approach could potentially provide new avenues for vaccine development [59].

It has been reported previously that the genetic system for antigenic variation in *M. bovis* is highly complex, mediated through *vsp* genes which undergo dynamic and spontaneous changes in size and expression leading to extensive sequence variations [13,40,60,61,62]. When we analyzed the presence of *vsp* genes in all the isolates using *vsp* genes (*n* = 13) from strain PG45 as a reference, all the isolates evaluated showed the absence of a complete set of *vsp* genes. However, a few genes, namely *vspJ*, *vspI*, and *vspK,* were detected in 73, 47 and 37 isolates, respectively. This suggests that of the *vsp* genes found in type strain PG45, *vspJ* is the most prevalent among other isolates of the species, followed by *vspI* and *vspK*.

In contrast to this, when we used the highly conserved 70 bp sequence present immediately upstream of the *vsp* gene start codons to identify a wider variety of *vsp* genes, we found 29/250 isolates possess ≥10 sequences, suggesting a high degree of distinctiveness in *vsp* genes at the sequence level. This massive variation in *vsp* sequences defines the vastly extended antigenic potential within the *M. bovis* isolates [60]. The persistence of this pathogen in variety of hosts and tissues suggests that it can easily adapt to environmental fluctuations as well as host defense mechanisms with the help of varied antigenic phenotypes [41]. This is in accordance with the results obtained in this study suggesting high degree of distinctiveness at *vsp* genes level. Not only the *vsp* genes diversity, but also the IS elements are playing a crucial role in defining the genome heterogeneity of *M. bovis*. Our results suggest that the IS elements are significantly increasing the genome size of *M. bovis* isolates and their average number in the complete genome was as high as 52.76, suggesting the distribution of these genes across the genomes. Therefore, further investigations are needed to analyze the genetic diversity of *vsp* genes in *M. bovis* isolates keeping in mind the possible roles of IS elements. Further, our analysis of genes associated with host type indicates that one *vsp* gene is significantly associated with bovine isolates, suggesting its possible role in adaptation of *M. bovis* in bovine hosts. Altogether, this analysis supports the inclusion of isolates with a high number of *vsp* genes in the autogenous vaccine.

## 5. Conclusions

Despite numerous advances in research pertaining to *M. bovis*, it remains a persistent threat to the cattle and bison industries. *M. bovis* is known to evade the host immune system through extensive antigenic divergence resulting from high recombination efficiency of its *vsp* genes. In addition, *M. bovis* is inherently refractory to a wide group of antibiotics due to lack of a cell wall. Therefore, in order to devise successful vaccines, it is important to understand the key genomic differences and the extent of diversity among *M. bovis* isolates. In this study, 147 genomes of genomes isolated from four different countries were sequenced and combined with publicly available *M. bovis* genome datasets in a first-ever large-scale comparative study of 250 *M. bovis* genomes. The analysis also focused on the host origin of the isolates to understand the *M. bovis* virulence-host association patterns. Our results revealed high divergence among isolates originating from the USA which clustered into five different clades based on single nucleotide polymorphisms (SNPs). On the contrary, strains from Australia were found to be minimally divergent and clustered within a single clade with all strains from China and two isolates from Israel. Although sampling bias is evident in the analyzed datasets, with USA isolates representing more than half (56%) of the isolates, the divergence of USA isolates could not be ruled out as one isolate, CG1-1544, clustered with the outgroup *M. agalactiae* 5632 revealing its highest ancestral homology. Our study showed that the clade IV comprised of all the bovine isolates, whereas clades III and V were dominated by bison isolates. Further, the clustering of isolates together from different hosts indicated the genomic flexibility of *M. bovis* strains for adaptation to different hosts. The analysis of pangenomic trends suggests a large diversity of *M. bovis* that yet remains to be explored. Analysis of genomic heterogeneity among *vsp* genes revealed that out of the 250 isolates, at least 29 harbor more than 10 *vsp* genes. In addition, a large number of IS elements that mediate recombination events was found in all of the *M. bovis* strains, suggesting high level of genomic rearrangements. Our results may also mean that the previously proposed vaccine candidates reported from bison may need to be revisited to include the broad functional diversity of *M. bovis* identified in our analysis.

## Figures and Tables

**Table 1:** Highest-ranking genes associated with host type (bovine vs bison).

Gene	Odds Ratio	*p*	Pairwise-p	Sensitivity	NCBI_Reference_Sequence
Hypothetical protein	8.351351	1.49E-10	0.0039–0.5078	58.1920904	WP_014829940.1
Lipoprotein	0.178279	1.48E-05	0.0117–0.2265	65.53672316	AEI90200.1
variable surface lipoprotein-4 (vspI)	3.564176	0.0005	0.0063–0.3876	34.46327684	WP_013456472.1
5-formyltetrahydrofolate cyclo-ligase	0.332237	0.0007	0.0390–0.5078	57.06214689	WP_013954957.1
hypothetical protein (ICEB-2 encoded)	4.974265	0.0008	0.0390–0.5078	23.16384181	WP_075271017.1
hypothetical protein	4.663043	0.0014	0.0019–0.1093	22.03389831	WP_075271089.1
putative transmembrane protein	0.403674	0.0019	0.0117–0.2265	29.94350282	WP_075271035.1
Lipoprotein	3.553957	0.0083	0.0019–0.3437	21.46892655	SBO46265.1
putative transmembrane protein	2.02886	0.0147	0.0390–0.507	64.40677966	AIA33704.1
lipoprotein	1.949627	0.0223	0.0224–0.2668	62.14689266	WP_075271416.1

## Data Availability

Raw genome sequence data for the *M. bovis* strains used in this study has been submitted under the bioproject PRJNA534329.

## Supplementary Materials

The following is available online at https://www.mdpi.com/2076-2607/8/10/1561/s1, Table S1. The metadata depicting the general genomic attributes of *M. bovis* isolates.
